# Children and parents’ perspectives on the acceptability of three management strategies for dental caries in primary teeth within the ‘Filling Children’s Teeth: Indicated or Not’ (FiCTION) randomised controlled trial – a qualitative study

**DOI:** 10.1186/s12903-020-1060-6

**Published:** 2020-03-12

**Authors:** Sarab El-Yousfi, Nicola P. T. Innes, Richard D. Holmes, Ruth Freeman, Kathryn B. Cunningham, Elaine McColl, Anne Maguire, Gail V. A. Douglas, Janet E. Clarkson, Zoe Marshman

**Affiliations:** 1 School of Clinical Dentistry, Claremont Crescent, Sheffield, S10 2TA UK; 2 School of Dentistry, University of Dundee, Park Place, Dundee, DD1 4HN UK; 3 School of Dental Sciences, Faculty of Medical Sciences, Newcastle University, Newcastle upon Tyne, NE2 4HH UK; 4 School of Medicine, University of St Andrews, North Haugh, St Andrews, KY16 9TF UK; 5 Population Health Sciences Institute, Newcastle University, Baddiley-Clark Building, Richardson Road, Newcastle upon Tyne, NE2 4AX UK; 6 University of Leeds School of Dentistry, Clarendon Way, Leeds, LS2 9LU UK; 7 Dental Health Services Research Unit, School of Dentistry, Park Place, Dundee, DD1 4HN UK

**Keywords:** Caries, Caries management, Qualitative, Children, Parents, Paediatric dentistry, Primary teeth, Primary dental care, Acceptability

## Abstract

**Background:**

The Filling Children’s Teeth: Indicated Or Not? (FiCTION) randomised controlled trial (RCT) aimed to explore the clinical- and cost-effectiveness of managing dental caries in children’s primary teeth. The trial compared three management strategies: conventional caries management with best practice prevention (C + P), biological management with best practice prevention (B + P) and best practice prevention alone (PA)-based approaches. Recently, the concept of treatment acceptability has gained attention and attempts have been made to provide a conceptual definition, however this has mainly focused on adults. Recognising the importance of evaluating the acceptability of interventions in addition to their effectiveness, particularly for multi-component complex interventions, the trial design included a qualitative component. The aim of this component was to explore the acceptability of the three strategies from the perspectives of the child participants and their parents.

**Methods:**

Qualitative exploration, based on the concept of acceptability. Participants were children already taking part in the FiCTION trial and their parents. Children were identified through purposive maximum variation sampling. The sample included children from the three management strategy arms who had been treated and followed up; median (IQR) follow-up was at 33.8 (23.8, 36.7) months. Semi-structured interviews with thirteen child-parent dyads. Interviews were transcribed verbatim and analysed using a framework approach.

**Results:**

Data saturation was reached after thirteen interviews. Each child-parent dyad took part in one interview together. The participants were eight girls and five boys aged 5–11 years and their parents. The children’s distribution across the trial arms was: C + P *n* = 4; B + P *n* = 5; PA *n* = 4. Three key factors influenced the acceptability of caries management in primary teeth to children and parents: i) experiences of specific procedures within management strategies; ii) experiences of anticipatory dental anxiety and; iii) perceptions of effectiveness (particularly whether pain was reduced). These factors were underpinned by a fourth key factor: the notion of trust in the dental professionals – this was pervasive across all arms.

**Conclusions:**

Overall children and parents found each of the three strategies for the management of dental caries in primary teeth acceptable, with trust in the dental professional playing an important role.

## Background

There has been a lack of evidence supporting effective management of dental caries in children’s primary teeth when treated in primary care, generating uncertainty for the dental profession and for parents and children around treatment planning [1–3]. To address this uncertainty, a pragmatic, multi-centre, three-arm, patient-randomised controlled trial set in NHS primary dental care was designed. The RCT, Filling Children’s Teeth: Indicated Or Not? (FiCTION) compared the incidence of dental pain and infection over a period of three years in 3–7 year-old children with at least one primary tooth with a carious lesion extending into dentine, when managed by one of three strategies [4]. The trial was conducted in dental practices and treatment provided by the child’s general dental practitioner (GDP) and team. Each dental practice was allocated to one of the trial’s five clinical centres: England (3), Scotland (1), and Wales (1).

The management strategies were:

1. Best Practice Prevention Alone (PA). This involved four components carried out according to current national guidelines: tooth brushing; dietary investigation, analysis and intervention; fissure sealants applied to permanent teeth; fluoride varnish applied to primary and permanent teeth. Within this arm there was no removal of carious tissue, no sealing-in of caries, and no restoration placement.
2. Conventional with Best Practice Prevention (C + P). This involved the administration of local anaesthetic (LA), complete removal of carious tissue mechanically, tooth preparation followed by placement of a restoration or preformed metal crown (PMC) to restore the tooth. Best practice prevention was carried out as above.
3. Biological with Best Practice Prevention (B + P). Carious tissue was either selectively removed and sealed into the tooth using an adhesive restorative material over the decay, or not removed at all and sealed with a fissure sealant or a PMC using the Hall Technique (the PMC was cemented in place without any local anaesthesia or tooth preparation). Best practice prevention was carried out as above.

Children with at least one primary molar tooth with a carious lesion into dentine were randomly allocated to one of the three strategies [4] and their caries managed according to that strategy, for up to three years. Throughout the trial, treatment was provided by a qualified dental professional (DP) such as a general dental practitioner (GDP), dental hygienist/therapist or dental nurse. All treatment was recorded by the DPs, including any deviations from the allocated treatment arm, i.e. items of treatment delivered outside the randomly allocated strategy.

The FiCTION trial included clinical, economic and patient-reported outcomes, which have been reported elsewhere [5, 6]. The trial also aimed to explore the acceptability of the treatment strategies for the DPs delivering the treatment [7] the children receiving the treatment and their parents. There is growing recognition of the importance of evaluating the acceptability of health interventions, in addition to their effectiveness [8]. This is particularly the case for multi-component complex interventions [9]. For successful implementation of an intervention, its clinical- and cost- effectiveness, as well as its acceptability, are important outcomes for both the healthcare professionals delivering the interventions and the patients receiving them [8]. Various attempts have been made to define and conceptualise treatment acceptability, including where different management strategies exist. For example, Sidani and colleagues described treatment acceptability as relating to ‘perceiving a treatment as favourable’ [10]. More recently, Sekhon and colleagues defined treatment acceptability as ‘a multi-faceted construct that reflects the extent to which people delivering or receiving a healthcare intervention consider it to be appropriate, based on anticipated or experienced cognitive and emotional responses to the intervention’ [8]. These attempts at conceptualising acceptability have, however, focused mainly on adults. Different management strategies are perceived to have variable levels of acceptability. These are related to treatment characteristics or attributes such as the level of invasiveness or need for injections, which individuals evaluate when deciding upon their preferences. The characteristics most commonly found to affect treatment choices are the effectiveness and appropriateness of the intervention in managing the clinical problem, as well as its convenience and suitability to the person’s lifestyle [8, 10].

The aim of this qualitative component of the FiCTION trial was to explore the acceptability of the three management strategies employed to manage decay in primary teeth, from the perspectives of children and their parents.

## Methods

A qualitative study employing exploratory semi-structured interviews with children participating in the FiCTION trial [4] and their parents was undertaken. An updated version of the FiCTION trial protocol is available at http://www.nets.nihr.ac.uk/projects/hta/074403. The East of Scotland Research Ethics Committee provided ethical approval for the trial, including the qualitative study (REC reference: 11/ES/0047). The Consolidated criteria for reporting qualitative research guidelines for reporting qualitative research were used [12] (Additional file 1).

### Participants

Towards the end of the trial children were selected from the list of all FiCTION participants who had not explicitly withdrawn from the trial. Parents of the children selected were also invited to participate. The sample was drawn from two of the five FiCTION clinical centres; one from Scotland and one from England. Children were identified from FiCTION trial records by means of purposive maximum variation sampling using the variables of sex, age, regional location and the dental practice in which the treatment was delivered. The sample included children from each of the three arms of the trial (C + P, B + P and PA), including children whose treatment deviated from the clinical protocol, as these were cases of particular interest.

For the purposes of this paper, the term “parent” represents parents or guardians.

### Recruitment and consent

Potential child-parent participants were initially sent a letter of invitation by their dental practices, which explained the aim and rationale of the study and what participation would involve. The researcher then contacted those who expressed an interest in participating to arrange a suitable time and location to hold the interview. There were no drop-outs by those who had registered interest. Before the start of each interview, the researcher obtained written informed consent from the parent and oral and written assent from the child, using age appropriate information sheets for the children. Recruitment continued until no new themes emerged from the interviews.

### Procedure

The median (IQR) follow-up period for all 1058 participants in the intention to treat (ITT) analysis set included in the FiCTION trial was 33.8 (23.8, 36.7) months. Each child-parent dyad took part in one interview that was conducted at the end of the child’s participation in the trial. The interviews were conducted in-person, by experienced qualitative doctoral researchers (ZM, KC, HC, JR) with different academic backgrounds, including dentistry and social science, and took place at each participant’s home or another convenient location. The interviewers included three females and one male and there was no relationship established with the participants prior to study commencement. To facilitate communication with children the interviews adopted participatory approaches (e.g. drawings, playing with Play-Doh dentist [Hasbro, Inc., Pawtucket, RI, USA], Dentist Barbie [Mattel, Inc., Segundo, CA, USA] or other dental toys). The topic guides were developed from literature on the concept of acceptability and its operationalisation [8] and through discussions with the FiCTION trial management group. The specific wording of questions was tailored to the participants and the topic guides were kept flexible, allowing for the discussion of unanticipated issues and their incorporation in subsequent interviews. The interviews lasted on average for 45 min and any field notes were made after the interview and were used to provide additional context to the analytical process. Interviews were audio-recorded and then transcribed verbatim and anonymised.

Parents were present for the child’s part of the interview. Each child and parent participating was given a shopping voucher and FiCTION-branded gifts to thank them for taking part.

### Data analysis

The transcribed interviews were analysed using Framework Analysis [11], chosen as it enabled the exploration of a priori themes identified from the literature search, while supporting an inductive approach by allowing for new themes to emerge. The discovery of new or unexpected concepts is a major strength of qualitative research. The framework approach is a matrix-based method for the analysis of cross-sectional qualitative data, involving the following stages:

1. Familiarisation
2. Identifying initial themes
3. Labelling the data
4. Sorting the data by theme
5. Synthesising the data

The data were primarily analysed by two dentally trained researchers with PhDs and experience of qualitative research (SE, ZM). Both researchers read and re-read all the transcripts to achieve familiarisation with the data and independently identified initial themes for discussion with the other authors. Codes were developed for categories relating to the themes that emerged. An analytic chart was then constructed with column headings for each category and a row for each interviewee. Transcripts were then systematically coded and relevant data was input into the charts with text referenced and linked to the source (by SE). This ensured that the researchers remained connected to the original raw data throughout the refinement stages and the text could be revisited to verify conclusions. This process continued as interviews progressed and themes emerged. The researchers met frequently throughout this process to discuss emerging themes, refine the framework and agree on final themes and sub-themes. Members of the wider research team were then debriefed and agreement between the researchers was reached through discussion and further review of the transcripts. This process strengthened inter-rater reliability and credibility and thus ensured the trustworthiness of the data analysis. Data were interpreted based on the research objectives and the inductively generated themes.

### Reflexivity

In research with children, efforts are required to minimise the power imbalance that exists between the researcher and the child [13]. In this study, this was addressed by ensuring that interviews took place where children were comfortable, using participatory activities and emphasising that children could stop the interview at any time [14]. The study was designed with input from a multi-disciplinary trial management group and the interviews were conducted by experienced qualitative interviewers who had an interest in children’s oral health and who were experienced at assessing signs of distress or boredom in participants, although none were observed.

## Results

Interviews were conducted with 13 child-parent dyads between January 2017 and November 2017. The children were aged from 5 to 11 years at the time of interview; eight girls and five boys from across the three trial arms: C + P *n* = 4; B + P *n* = 5; PA *n* = 4.

Participants had been treated by six dental teams and treatment deviations had occurred for four participants. Three parents were male and ten were female (Table 1).

**Table 1:** Table of participants

Code	Participant	Trial arm	FICTION clinical centre
LC01	Child	PA	England
LP01	Parent		
LC02	Child	C + P	England
LP02	Parent		
LC03	Child	C + P	England
LP03	Parent		
LC04	Child	B + P	England
LP04	Parent		
LC05	Child	B + P	England
LP05	Parent		
LC06	Child	B + P	England
LP06	Parent		
LC07	Child	B + P	England
LP07	Parent		
LC08	Child	C + P	England
LP08	Parent		
SC01	Child	B + P	Scotland
SP01	Parent		
SC02	Child	PA	Scotland
SP02	Parent		
SC03	Child	PA	Scotland
SP03	Parent		
SC04	Child	PA	Scotland
SP04	Parent		
SC05	Child	C + P	Scotland
SP05	Parent

**Table 2:** Key factors influencing acceptability of the three management strategies from the perspectives of participants

Key factors influencing acceptability	
**Trust in dental practitioner providing care**	**Experiences of specific procedures based on the management strategy:**
	*Conventional with best practice prevention*
	*Biological with best practice prevention*
	*Best practice prevention alone*
	**Anticipatory dental anxiety**
	**Perceptions of effectiveness**

Four factors were found to influence the acceptability of caries management in primary teeth for children and parents. These included their: 1) experiences of specific procedures based on the management strategy; 2) anticipatory dental anxiety; 3) perceptions of effectiveness in terms of pain relief and improved oral health. These three factors were underpinned by another key factor; the notion of trust in the DP and the development of a treatment alliance. This notion was pervasive, regardless of which treatment strategy the child had been randomised to  (Table 2).

### Experiences of specific procedures

Overall, while there was some variation between dyads and between specific dental procedures delivered by different DPs, the procedures delivered within all three strategies were found to be generally acceptable to both children and parents.

### Conventional with best practice prevention (C + P)

The C + P arm was described by some parents as the ‘traditional’ or ‘expected’ treatment based on their own experience; they were not aware of other options.

*I think it was the filling, the traditional filling … ..I didn’t realise there were other treatment options to be fair. I had to get a filling, it was a needle … … and I expected it before I was told about any other options*. (SP05: parent, Scotland).

Children reported their dislike for some specific dental procedures within this strategy, namely local anaesthetic (LA) injections, extractions, tooth preparation and the physical conventional placement of pre-formed metal crowns (PMC). Children’s descriptions of what they disliked the most about the dental visit included:

*when he numbs your teeth …*. *Because it feels like your lips are about three-and-a-half miles long*. (LC08: child, England).

*Well … when they put the sleepy juice inside.* (LC03: child, England).

*It was that bit of having my teeth out that wasn’t a nice bit. They use tweezers … Feels like it’s still in but it’s bendy a bit … I just feel it … ..it feels like a bit of pushing and pulling.* (LC02: child, England).

*She did something with this drill … It’s a little silver crown that went over the tooth … They felt a bit weird … yeah they feel a bit fizzy … they don’t feel fizzy now it’s just when they go on.* (LC02: child, England).

Some parents viewed the more invasive nature of the C + P approach for managing carious primary teeth negatively.

*I wondered why they were gonna put her through that when this thing was going to fall out anyway …*. *why are you filling a tooth that’s gonna just fall out*? (SP05: parent, Scotland).

Past dental experiences affected parents’ preferences and expectations of fillings and injections and it appeared that some parents re-lived their own childhood dental experiences when their child attended for dental treatment.

*I probably was more upset than her because I remember the pain of it myself … .would have preferred if she didn’t get to have a big needle in her mouth* (SP05: parent, Scotland).

*…*. *when I was little I was petrified because I had a bad experience. And obviously because my dentist weren’t as good as what they got now, I ended up losing my teeth on bottom...once you have a bad experience it puts you off it a little bit*. (LP08: parent, England).

Parents raised an additional concern regarding the aesthetic aspect of PMCs in a child’s mouth as potentially being a visible sign of inadequacies in their parenting practices, but felt that if it was the best option then it was justified.

*Obviously, I’m to blame as the Mum for the overall hygiene of his teeth. But you just felt like okay, everyone would see how bad mum I am … … But then I thought you know what, whatever is best for you. That’s what I was going to do*. (LP02: parent, England).

On the other hand, children were not concerned with the aesthetics of PMCs and were excited to show them to others.

*Like the whole class. And my next door neighbour. And she’s got three …*. *They said, “Oh, he’s got a silver tooth. Did they pull it out and put it in?” … .. I like it*. (LC03: child, England).

Despite voicing some concerns regarding specific procedures, children found it possible to have the dental treatment procedures and would be willing to undergo them again. Parents found the C + P approach acceptable and this was strongly associated with trust in the DP providing treatment. This trust enabled a treatment alliance between the child, parent and DP and facilitated the acceptance of the treatment by the child.

*Well after … the first one* [extraction]*, I felt a bit scared, but the second one* [extraction]*, I knew that I’d already done it.* (SC05: child, Scotland).

*Not nice … .. Well, it was kind of nice … Because it was my third time coming. And then, I kept really, really still. It* [the injection] *didn’t hurt.* (LC03: child, England).

*At some point it felt like it hurt a little bit, but if you get used to it and you just get on with it, it’ll be fine.* (LC08: child, England).

*It’s a bit painful when they do that, injection. That’s why he was a little bit scared. But he was fine. I was very happy with everything.* (LP03: parent, England).

*He’s absolutely fantastic …*. *he always does numb it before he puts the needle in, to be fair. And I have never known a dentist do that.* (LP08: parent, England).

### Biological with best practice prevention (B + P)

Children and parents found the B + P strategy acceptable and reported that they would agree to undergo the procedure again if needed. Parents also expressed the value they placed on avoiding any drilling and injections.

*Say she had another hole and that … .had to happen again … .. how would you feel?* (Interviewer).

*Oh yeah it’d be fine … … I’d take her straightaway I’d feel a lot more at ease now. It won’t bother you neither would it babes?* (LP06: parent, England).

*No* (LC06: child, England).

*So if your mum said you would have to go tomorrow you would be fine?* Interviewer.

*Yeah* (LC06: child, England).

*Well, I felt good with this one … ...because one of the other ones has got some injections in it.* (LP04: parent, England).

*I’m very glad she got this one …*. *if someone came near her mouth with the drill, she wouldn’t be happy at all.* (LP05: parent, England).

*It’s better that than a filling and drilling and injections*. (SP01: parent, Scotland).

As with the C + P strategy, children reported their dislike of specific procedures, such as removal of some carious tissue and the actual fitting of a PMC using the Hall Technique; the latter was disliked due to the pain or discomfort that resulted from the pressure applied when placing it or if it was the wrong size.

*They’re like trying to clean it out …*. *It hurt a bit and I also felt a bit weird. Sort of like feeling like you haven’t felt anything like that before* (LC06: child, England).

*… she weren’t too keen on when they were cleaning her teeth you know the little metal thing that they’re putting in. I think it was just the tugging and the noises she could hear and feel that put her off a bit. But other than that, no she, we were fine with everything.* (LP06: parent, England).

*Quite sore …*. *He made sure it fit your tooth and then he put it on …*. *It’s just stinging when he tries to fit it on*. (SC01: child, Scotland).

*The first one as I say, he did say this was the wrong size. She wouldn’t cry in front him … she cried when she got out …*. *She said it was sore*. (SP01: parent, Scotland).

*But I didn’t cry the second time*. (SC01: child, Scotland).

*No, the second time was fine*. (SP01: parent, Scotland).

*That’s the bit you don’t like …*? (LP05: parent, England).

*Oh the pushing?* (Interviewer).

*Yep*. (LC05: child, England).

The ‘unnatural’ aesthetics of a PMC, similar to when they were used in the C + P arm, was a concern for some parents. There was concern that their child may become self-conscious about their appearance.

*Slightly worried that with … all of her back teeth capped now … that like she’d notice, that other children didn’t … But she’s been absolutely fine. She’s not bothered by it*. (LP05: parent, England).

Some parents preferred a more restorative approach over Prevention alone (PA) however they had reservations regarding the ‘unnatural’ appearance of PMCs.

*I’d have been a bit iffy about probably leaving it and waiting and seeing, but I’m not quite sure how I feel about the stainless steel thing to be quite honest with you. I think I’d have preferred them to try and fill it rather than … you see it looks more natural*. (LP06: parent, England).

Conversely, some parents did not have any aesthetic concerns.

*It seemed sensible …*. *don’t really care what the look of it is*. (SP01: parent, Scotland).

Children did not express any concern regarding the aesthetics of PMCs and parents spoke of their child showing them off.

*She had a question asked about her silver crowns and one of her friends liked it and wanted it … ..she was showing off with them.* (LP04: parent, England).

Acceptability of procedures was once again linked to trust and the building of a treatment alliance between the child, the parent and the DP providing care. The patient management skills of the DP were able to facilitate a trusting relationship which had a positive impact on compliance and reducing dental anxiety in both child and parent.

*When before appointments, she was crying and everything, but when the dentist was suggesting us to do it in front of her and she was listening to her, and then when we’re coming and she was saying, okay, you’re allowed to do it just because of the dentist*. (LP04: parent, England).

*She’s not frightened of the dentist at all. You go in and they’re lovely …*. *They make you feel like you’re at home*. (LP06: parent, England).

### Best practice prevention alone (PA)

Children and parents expressed contentment with being allocated to the PA approach as this avoided having invasive procedures such as restorations and injections. Some parents were also content to avoid having PMCs for the previously mentioned aesthetic reasons. Parents, however, raised concerns about the potential of further deterioration of their child’s teeth resulting in dental pain and/or affecting the child’s permanent successor tooth. Trusting the DP to make the right decision was a significant factor in parents’ acceptability of this arm.

*I’m all for that provided it doesn’t cause any more damage … My two concerns were A) … the decay was going to cause more damage and therefore she’s going to get some pain from it. And the second thing is whether it’s going to damage the adult teeth underneath … the fact I trust [DP name] …*. *She’s very clear, she explains things very … and really takes the time both with [child’s name] and me …*. *And that helps I think to make a decision*. (LP01: parent, England).

Parents in the PA route found this strategy acceptable as long as the carious teeth were pain-free and considered it the less ‘radical’ or ‘significant’ method of treatment.

*But none of the three are causing her any pain. I think that’s the key thing for me. So we’re trying to do this thing with diet and brushing, with full strength toothpaste and all that kind of things.****… …****I think that it’s all down to pain. So that would obviously influence that decision*. (LP01: parent, England).

*If she’d have been having a lot of pain, I’d have thought differently and I think … something more radical to either get rid of it, take the tooth out or to have a filling or whatever, so do something more significant rather than just the painting*. (LP01: parent, England).

Parents preferred avoiding fillings and found other aspects of the PA arm a positive experience.

*Obviously, if he doesn’t have to get treatment then, we would rather it wasn’t …*. *if he doesn’t need then, I don’t want them doing it*. (SP02: parent, Scotland).

*I’d say a lot of positive things has come out of it. There’s nothing negative, definitely something positive. And it makes the children aware …*. *what they’re eating and what they’re doing...I think it’s been really helpful*. (SP04: parent, Scotland).

Their preferences also appeared to be affected by personal past experiences.

*I prefer the preventative, because I’ve had fillings and I didn’t like getting them at all. And that kind of puts the fear in. So, if we can stop getting fillings, then, we can stop the fea*r. (SP03: parent, Scotland).

*I was kind of freaking out, it was such a relief when she said that the fluoride could control it. It was such a relief. Obviously, I’ve done it myself and it’s just not a nice thing. Just terrible.* (SP02: parent, Scotland).

Children and parents had different views regarding the aesthetics of PMCs. Some parents disfavoured PMCs for aesthetic reasons and thus favoured this approach.

*A silver cap on that tooth but we’ve at the moment decided not to*. (LP01: parent, England).

*I want one*. (LC01: child, England).

*She wants one, I’m not sure. I think it’s me that’s saying no*. *I just … well, partly the aesthetics. I think having a piece of lump of silver in her mouth is not ideal at this age*. (LP01: parent, England).

*It is*. (LC01: child, England).

*I mean, she sees silver crown and thinks different to what I think I guess.* (LP01: parent, England).

Parents also reported that, in addition to their child’s experience, they found the PA strategy to be beneficial to them as a parent in terms of encouraging ways to reduce sugar consumption and improve tooth brushing.

*… ..and just like small bits of information that I never really knew …*. *Probably about the tomato sauce actually. That really surprised me. Just the amount of sugar actually in it.* (SP04: parent, Scotland).

*… ..spent quite a lot of time on helping us to brush properly …*. *she’s very good in terms of giving us advice in terms of how to brush and obviously looking at the pink and knowing where we’re missing, that helps as well.* (LP01: parent, England).

In summary, the procedures delivered within all three of the management strategies were generally acceptable to children and parents.

### Anticipatory dental anxiety

Children and parents in all three arms reported being anxious at the thought of certain procedures such as drilling, injections and extractions. Even in children with positive experiences, the thought of certain procedures provoked a feeling of dislike associated with dental visits.

For example, the following 6-year-old girl had mixed feelings about attending a dental visit. The child explains that the reason for being ‘grumpy’ about the dental visit is the dislike of having her teeth extracted despite not having any actual prior extraction experience.

*I don’t know. Sometimes I’m a bit grumpy and sometimes I’m really happy*. (LC05: child, England).

*What is it about coming to the dentist that makes you grumpy?* (Interviewer).

*I don’t know*. (LC05: child, England).

*Is there something that the dentist does that makes you a bit grumpy?* (Interviewer).

*Pulls my teeth out*. (LC05: child, England).

S*he’s never pulled your teeth out.* (LP05: parent, England).

This anticipatory dental anxiety was common amongst children who had not experienced tooth extraction and reported being worried it.

*I don’t like to take my tooth out.* (LC04: child, England).

*No?* (Interviewer).

*It will make me a bit worried.* (LC04: child, England).

*What is it that would make you worried?* (Interviewer).

*I don’t like to take my tooth out. I just really like to pull my teeth out when it already falls over.* (LC04: child, England).

*You don’t want to have a tooth taken out?* (Interviewer).

*No* (LC04: child, England).

*But you’ve not had one taken out?* (Interviewer).

*Yeah, I didn’t have one out.* (LC04: child, England).

*I sometimes get scared, if like she says get a tooth taken out. I sometimes get scared if that’s going to happen.* (SC03: child, Scotland).

*But … you’ve never had a tooth taken out at the dentist. I think it’s just a general worry. Her teeth have all come out naturally …* (SP03: parent, Scotland).

The prospect of having injections also brought on feelings of anxiety. When asked about how she felt about having holes in her tooth and having to visit the dentist, this child replied:

*Probably okay until I found out I had to get a needle.* (SC05: child, Scotland).

Being anxious of certain procedures because of potential pain, and concern that their child would not cooperate, was also reported by parents.

*I think I were more nervous at first then she were … It was just the thought of her having an injection I thought, oh no it’s going to hurt … She’s not going to let them do it. But no, she were fine … ..he talked her through it. No problems at all*. (LP06: parent, England).

Some felt that when their child had prior knowledge of the specific procedures to be undertaken at the next dental visit that this would increase anxiety.

*I think if they’re told they’re going for something, they get more worried … because I think if I’d just said we’ve got another check-up and then when she was in the chair, that would save her because she gets worked up way in advance.* (SP05: parent, Scotland).

Parents also reported being concerned about their child’s willingness to return for certain procedures.

*And she’s playing with the drill [toy] but, like, if someone came near her mouth with the drill, she wouldn’t be happy at all. And I think we’d have had a lot more problems in getting her to sit down and keep coming back*. (LP05: parent, England).

Despite anticipating their child’s non-cooperation, parents found that the dental team was usually able to facilitate the child’s acceptance of the procedure. Trust in the DP providing care was a significant factor in managing dental anxiety. Parents reported that their child’s cooperation with treatment was positively affected by the skills of the DP: their ability to make children feel comfortable and less anxious was important for parents.

*We’re very lucky because she really, really likes ‘the dentist’, don’t you? …*. *She’s made you feel really, really comfortable. Sometimes even if when you’re feeling a bit nervous, she’ll still get in the chair and at least let her look and things*. (LP05: parent, England).

*… I thought she would be pulling away from them, but she just let them get on and she was okay.* (SP05: parent, Scotland).

*She likes [DP name] so much … .so she wants to be there all the time she said as well … And she’s quiet as well when she gets this treatment done.* (LP04: parent, England).

*I suppose I would be more concerned if she’d then said, “I don’t want to go back.” But no, she’s been fine. They like him and he’s nice.* (SP01: parent, Scotland).

### Perceptions of effectiveness (in terms of reduced pain and improved oral health)

Children and parents across all strategies reported a positive impact in terms of less pain and improved oral health. Parents felt it was important that, when their child was in pain, it was quickly relieved and efforts were made to prevent repeat episodes.

*Yes, it seems fine. And we have no complaints, no problems, no toothaches with it …*. *she doesn’t complain when she’s eating or anything anymore*. (LP05: parent, England).

*She was about three [years of age] when we came first to the dentist, I was very worried because …*. I *didn’t want her to be ill with these holes. But nothing worries me now because it did improve and like it’s been repaired*. (LP04: parent, England).

Parents reported an overall improvement in their child’s oral health with changes in their oral health behaviours, including improved tooth brushing and reduced sugar intake.

*There’s a massive change in his teeth*. (LP02: parent, England).

*And about fizzy drinks, they’re all quite conscious about they’ll go “Oh, [DP name] says but only as a treat.” [DP name] does it in a nicest possible way, but in a way that they remember so. And it’s good for them especially as they’re getting older.* (SP04: parent, Scotland).

*And, then she was not letting us to brush her teeth, it was a really big problem, and this improved now as well … because of the dentist.* (LP04: parent, England).

Some children also reported changes in their oral health behaviour and gaining oral health knowledge.

*He’s taught me how to brush my teeth … I didn’t know*. (LC07: child, England).

*Less unhealthy food … fat … sugar … and fizzy juice...I’ve started to cut down on it*. (SC05: child, Scotland).

*Kind of stops me from eating so much. So, I find I eat more healthier.* (SC03: child, Scotland).

Parents who trusted the DP were confident that their child was being well cared for; they spoke of their trust in their own personal DP and related this to their acceptability and trust of their child’s DP in the FiCTION trial. Thus, the treatment alliance may be built between the DP and the child through the personal beliefs and experiences of the parent.

*Basically, I left everything to the dentist. He knows what he’s doing and he’s brilliant; he’d do whatever he could and do his best in his power. So I trusted his decision and choices*. (LP06: parent, England).

*She knows what she’s doing and she takes care of my teeth … I mean my teeth were a disgrace when I went to her. And she’s fixed them all up and I’ll just go and say, “What do you think?” And kind of whatever she says, I’ll go with it*. *If she was to say, “Oh yeah it needs to come out, it’s going to cause a problem,” then we would do it.* (SP02: parent, Scotland).

### Trust in the DP providing care

Trust and the building of the treatment alliance was a significant factor in the acceptability of all management strategies as has been illustrated throughout this paper. Continuity of care appeared to be particularly important to allow trust to be built up. Child participants were co-operative and less anxious when they trusted the DP, suggesting a treatment alliance was achieved. Child-parent dyads spoke about the importance of their relationship with the DP and how being able to trust the DP resulted in a more acceptable experience. Both children and parents described their positive experiences with the DP and spoke of listening, explaining procedures, being gentle, caring, and patient as important characteristics in a DP.

Continuity of care with the same DP and regular visits was important in allowing the DP to gain the trust of the child and parent.

*She’s very patient with [child’s name], particularly with the children in helping her to understand. When she first came she was very reluctant to even open her mouth*. **… … … ..***We’ve been coming here forever***.** (LP01: parent, England).

*She’s got used it. She’s more comfortable with her surroundings and she knows what to expect. She’s a lot happier and a lot more settled.* (LP06: parent, England).

Empathy shown by the DP when caring for their child, was important for developing the treatment alliance and having a positive impact on acceptability.

*That my dentist is really gentle … she uses the tools pretty gently and she doesn’t like grab your gum like grabs up, but she does it really gentle … … I actually like it now because I thought she was going to be like really rough, but she’s actually really gentle.* (SC04: child, Scotland).

*Yeah, and he’s fantastic with him*. (LP08: parent, England).

*He’s pretty good. He’s just good at giving advice and everything like that.* (LC08: child, England).

*He’s caring isn’t he, as well?* (LP08: parent, England).

*If he wasn’t happy, he would cry. I would know if he’s unhappy or he’s upset. So he’s never, yeah. … … She’s good with him. And he kind of like listens so... It’s her who speaks to him gently and he is listening and yeah.* (LP02: parent, England).

Empathy was described as an attribute of a ‘good’ DP. Conversely, a DP that was ‘not good’ lacked empathy when caring for their patient.

*Shows concern. it helps if you’ve got a bit of time; ask them what they’re doing at school.* (LP07: parent, England.

*It’s just that he was a bit short with them and they hadn’t a ton of patience*. (LP07: parent, England.

Engaging with parents personally, allowing them the opportunity to support their child while undergoing treatment was also appreciated.

*[DP name] explains everything really well. And like she’s very big on pull a chair up, hold her hand, have a look at what we’re doing*. (LP05: parent, England).

## Discussion

Each of the three management strategies was a multi-component intervention to manage carious lesions and attempt to prevent more decay developing. Generally, all three arms were felt by children and their parents to be acceptable, with trust in the DP playing a significant role. Certain procedures, including LA and extractions, were more likely to be viewed negatively. Other associated factors identified by children and parents were anticipatory dental anxiety and perceptions of effectiveness. Children and parents had similar perspectives on most aspects of the management of carious tooth tissues except with respect to the PMC. Some parents were concerned about the aesthetics of PMCs (whether this was placed using a conventional technique or the Hall Technique); however, children did not share this view. This highlights the need for inclusion of both children’s and parents’ perspectives in future research assessing acceptability of management strategies.

The characteristics of acceptability previously described in the literature relating to adults, including perceived effectiveness and appropriateness in managing the clinical problem, were observed in our data. Some children and parents mentioned specific procedures within the multi-component intervention that they disliked, however the overall management strategies were still viewed as acceptable. As the definition of acceptability provided by Sekhon and colleagues suggested, views on both anticipated and experienced responses to the intervention were described [8].

The clinical and patient management skills of DPs influenced the establishment of trust and the treatment alliance between the clinician and children and their parents. Once this was established, DPs were able to reduce anticipatory dental anxiety and increase acceptability of the intervention for both child and parent. These observations may be formulated as the treatment alliance in which the trusting relationship between parent and DP and the ability of the parent and DP to contain the child’s worries and concerns, enables the child to accept the treatment that is being offered. These findings are in agreement with a study highlighting the importance of the parent in facilitating the child’s acceptance of treatment [15]. Child-parent dyads described positive experiences with their DP and mentioned listening, empathy, and patience as important characteristics in a DP. This is in line with a study reporting the importance of affective communication in facilitating and maintaining the treatment alliance between DP, child and parent [16]. This highlights the importance of the personal and professional characteristics of the provider and suggests that evaluation of the acceptability of the interventions should not be viewed in isolation from these.

Evaluating the acceptability of each of the management strategies in the FiCTION trial was important and allowed triangulation with the trial findings for the other outcomes. The trial found no evidence of a difference between arms in its primary outcome; dental pain and/or dental infection, nor were any differences detected in the trial’s secondary outcomes of caries incidence, child oral health-related quality of life or dental anxiety between the three caries management strategies [5, 6]. When acceptability of the management strategies was evaluated from the perspective of the DPs providing the treatment, they described their responsibility to select an appropriate strategy for each individual child, based on discussions with the child and parent and their own clinical experience [7]. Consequently, the trial recorded cross-arm deviations for 6% of the total visits. Cross-arm deviations were due to various factors, relating to the child – such as child pre-cooperative for LA – (20.6%), child anxiety (11.3%), and other child factors (not anxiety/cooperation, 3.8%) – the DP’s clinical judgement (29%) and parent’s wishes (28%). Together, the clinical effectiveness and acceptability findings suggest best practice as requiring a more complex approach to the management of caries in children’s primary teeth, involving a process of shared decision-making and taking into account the whole spectrum of child, parent and DP factors.

There were several limitations of this qualitative study. Firstly, it was not possible to embed the qualitative component throughout the FiCTION trial. Median (IQR) follow-up was at 33.8 (23.8, 36.7) months therefore children were interviewed 2–3 years after their treatment, rather than concurrently with intervention delivery. This may have affected participant’s responses. Secondly, only the perspectives of those who were retained within the trial were gained. It was not possible to interview children and parents who had withdrawn from the trial. Thirdly, the sample included only two regions (England and Scotland). Finally, during the interviews with children and parents, it was difficult to distinguish between procedures children had received during the trial from those before or since, especially if the child had received treatment to their permanent teeth or treatment outside of the trial after their time in the trial ended, although this is unlikely to alter the findings about the acceptability of the management strategies under investigation.

## Conclusion

Children and their parents found each of the three strategies for the management of dental caries in primary teeth acceptable. An exploration of the acceptability of interventions under investigation in a trial is invaluable in complementing the outcome evaluation.

## Supplementary information

**Additional file 1.** Consolidated criteria for reporting qualitative studies (COREQ): 32-item checklist.

## Data Availability

The datasets analysed in the current study are available from the corresponding author on reasonable request.

## Abbreviations

B + P: Biological management of caries with best practice prevention
C + P: Conventional management of caries with best practice prevention
DP: Dental professional
FiCTION: Filling Children’s Teeth: Indicated Or Not?
GDC: General Dental Council
GDP: General dental practitioner
LA: Local anaesthetic
PA: Best practice prevention alone
PMC: Preformed metal crown
RCT: Randomised controlled trial

## References

[1] Fayle SA, Welbury RR, Roberts JF (2001). British Society of Paediatric Dentistry: a policy document on management of caries in the primary dentition. Int J Paediatr Dent.

[2] Levine RS, Pitts NB, Nugent ZJ (2002). The fate of 1,587 unrestored carious deciduous teeth: a retrospective general dental practice based study from northern England. Br Dent J.

[3] Tickle M, Milsom K, King D, Kearney-Mitchell P, Blinkhorn A (2002). The fate of the carious primary teeth of children who regularly attend the general dental service. Br Dent J.

[4] Innes NP, Clarkson JE, Speed C, Douglas GVA, Maguire A, Fi CTC (2013). The FiCTION dental trial protocol – filling children’s teeth: indicated or not?. BMC Oral Health.

[5] Innes, NP, Clarkson, JE, Douglas, GVA, Ryan, V, Wilson, N, Homer, T, Marshman, Z, McColl, E, Vale, L, Robertson, M et al Child caries management: a randomized controlled trial in dental practice. J Dent Res 2019, 0(0):0022034519888882. 10.1177/0022034519888882 31771385

[6] Maguire A, Clarkson JE, Douglas GV, Ryan V, Homer T, Marshman Z, McColl E, Wilson N, Vale L, Robertson M, et al. Best-practice prevention alone or with conventional or biological caries management for 3- to 7-year-olds: the FiCTION three-arm RCT. Health Technol Assess. 2019;23. 10.3310/hta24010 PMC6983909 31928611

[7] Marshman Z, Kettle JE, Holmes RD, Cunningham KB, Freeman R, Gibson BJ, McColl E, Maguire A, Douglas GVA, Clarkson JE et al. Dental professionals’ experiences of managing children with dental caries in their primary teeth within the ‘Filling Children’s Teeth: Indicated Or Not’ Randomised Controlled Trial – a qualitative study BMC Oral Health. 20:64. 10.1186/s12903-020-1051-7. 10.1186/s12903-020-1051-7 PMC7057668 32131801

[8] Sekhon M, Cartwright M, Francis JJ (2017). Acceptability of healthcare interventions: an overview of reviews and development of a theoretical framework. BMC Health Serv Res.

[9] Moore GF, Audrey S, Barker M, Bond L, Bonell C, Hardeman W, Moore L, O'Cathain A, Tinati T, Wight D (2015). Process evaluation of complex interventions: Medical Research Council guidance. Bmj.

[10] Sidani S, Epstein DR, Bootzin RR, Moritz P, Miranda J (2009). Assessment of preferences for treatment: validation of a measure. Res Nurs Health.

[11] Spencer L, Ritchie J, O'Conner W, Ritchie J, Lewis J (2003). Analysis: practices, principles and processes. qualitative research practice.

[12] Tong A, Sainsbury P, Craig J (2007). Consolidated criteria for reporting qualitative research (COREQ): a 32-item checklist for interviews and focus groups. Int J Qual Health Care.

[13] Marshman Z, Gupta E, Baker SR, Robinson PG, Owens J, Rodd HD, Benson PE, Gibson B (2015). Seen and heard: towards child participation in dental research. Int J Paediatr Dent.

[14] Marshman Z, Hall MJ (2008). Oral health research with children. Int J Paediatr Dent.

[15] Freeman R (1999). The psychology of dental patient care: the case for mother* in the surgery. Br Dent J.

[16] Freeman R (2008). Communicating with children and parents: recommendations for a child-parent-centred approach for paediatric dentistry. Eur Arch Paediatr Dent.

